# Screening of core genes prognostic for sepsis and construction of a ceRNA regulatory network

**DOI:** 10.1186/s12920-023-01460-8

**Published:** 2023-02-28

**Authors:** Qian Zhang, Chenglin Wang, Shilin Li, Yang Li, Muhu Chen, Yingchun Hu

**Affiliations:** 1 Department of Infectious Diseases, The Affiliated Hospital of Southwest Medical University, Luzhou, Sichuan China; 2 Department of Emergency Medicine, The Affiliated Hospital of Southwest Medical University, Luzhou, Sichuan China

**Keywords:** Sepsis, Prognosis, RNA-seq, Messenger RNA (mRNA), MicroRNA (miRNA), Long non-coding RNA (lncRNA), Competing endogenous RNA (ceRNA)

## Abstract

**Objective:**

To screen out core genes potentially prognostic for sepsis and construct a competing endogenous RNA (ceRNA) regulatory network.

**Methods:**

Subjects included in this project were 23 sepsis patients and 10 healthy people. RNA-seq for lncRNA, miRNA and mRNA was performed in the peripheral blood samples. Differentially expressed RNAs (DER) were screened out for further analysis. GO annotation and GSEA functional clustering were performed to view the functional enrichment of DEmRNAs. Core genes of prognostic significance were screened out with the weighted correlation network analysis (WGCNA). Meta-analysis and Survival analysis was devised in different microarray datasets. RT-qPCR was conducted to validate these core genes. A ceRNA network was accordingly constructed according to the correlation analysis and molecular interaction prediction.

**Results:**

RNA-seq and differential analysis screened out 1,044 DEmRNAs, 66 DEmiRNAs and 155 DElncRNAs. The GO and GSEA analysis revealed that DEmRNAs are mainly involved in inflammatory response, immune regulation, neutrophil activation. WGCNA revealed 4 potential core genes, including CD247, IL-2Rβ, TGF-βR3 and IL-1R2. In vitro cellular experiment showed up-regulated expression of IL-1R2 while down-regulated of CD247, IL-2Rβ, TGF-βR3 in sepsis patients. Correspondingly, a ceRNA regulatory network was build based on the core genes, and multiple lncRNAs and miRNAs were identified to have a potential regulatory role in sepsis.

**Conclusion:**

This study identified four core genes, including CD247, IL-1R2, IL-2Rβ and TGF-βR3, with potential to be novel biomarkers for the prognosis of sepsis. In the meantime, a ceRNA network was constructed aiming to guide further study on prognostic mechanism in sepsis.

**Supplementary Information:**

The online version contains supplementary material available at 10.1186/s12920-023-01460-8.

## Introduction

Sepsis is a type of life-threatening organ dysfunction caused by a dysregulated host response to infection, and it is one of the most critical issues in the Modern Medicine [1, 2]. According to statistics, approximately 31.5 million patients are admitted for sepsis annually in the world, including 5.3 million deaths [3]. Increasing anti-inflammatory drugs are being available in treatment for sepsis, but the efficacy remains limited [4]. Besides, the lack of diagnostic specificity of the disease results in a slow progress of relevant clinical study and low diagnostic accuracy without a gold standard for diagnosis [5]. Given that sepsis is defined as a medical emergency that poses a threat to life, early diagnosis and timely treatment are critically important in susceptible persons [6], and potential biomarkers and molecular therapeutic targets specific to sepsis require to be identified.

Ideally, biomarkers are capable of differentiating between bacterial infection and other non-infectious systemic inflammation, which could be fast and reliable. In that way, sepsis could be recognized in early stages, and it may help for risk stratification, prognostic assessment and decision-making for use of antibiotics [7]. Identification of molecular targets prognostic for sepsis might be key for development of new treatment strategies [8].

A variety of transcription factors have shown a key part in the pathophysiological processes after sepsis, such as nuclear factor kappa-B (NF-κB) and activator protein-1 (AP-1), through induction of the expression of multiple related genes and products. Under this background, sepsis is also regarded as a genic disorder and gene therapy is emerging as a novel treatment approach [8]. Upon sepsis onset, high- and low-inflammatory responses occur simultaneously in repose to dysregulated gene expression. Reprogramming for pro- and anti-inflammatory genes, and the immune response genes involved in systemic acute inflammation, therefore, is also an approach that can prevent organ failure and improve outcome in sepsis [9]. Messenger RNA (mRNA) and non-coding RNA (ncRNA) are the involved transcripts, and mRNA expression profiling has been prevalently studied. Other RNAs, such as microRNA (miRNA), long non-coding RNA (lncRNA), circular RNA (circRNA), have also been extensively researched for the past few years [10]. Competing endogenous RNA (ceRNA) is receiving increasing attention with the development of the mechanism of post-transcriptional regulation. ceRNA is a pattern of regulating gene expression via competitively binding to a common miRNA response element with target mRNA at the post-transcriptional level [11, 12]. Recent research suggested that ceRNA is highly implicated in tumorigenesis and development [13]. However, the specific ceRNA regulatory mechanism in sepsis remains to be fully understood.

**Fig. 1:**
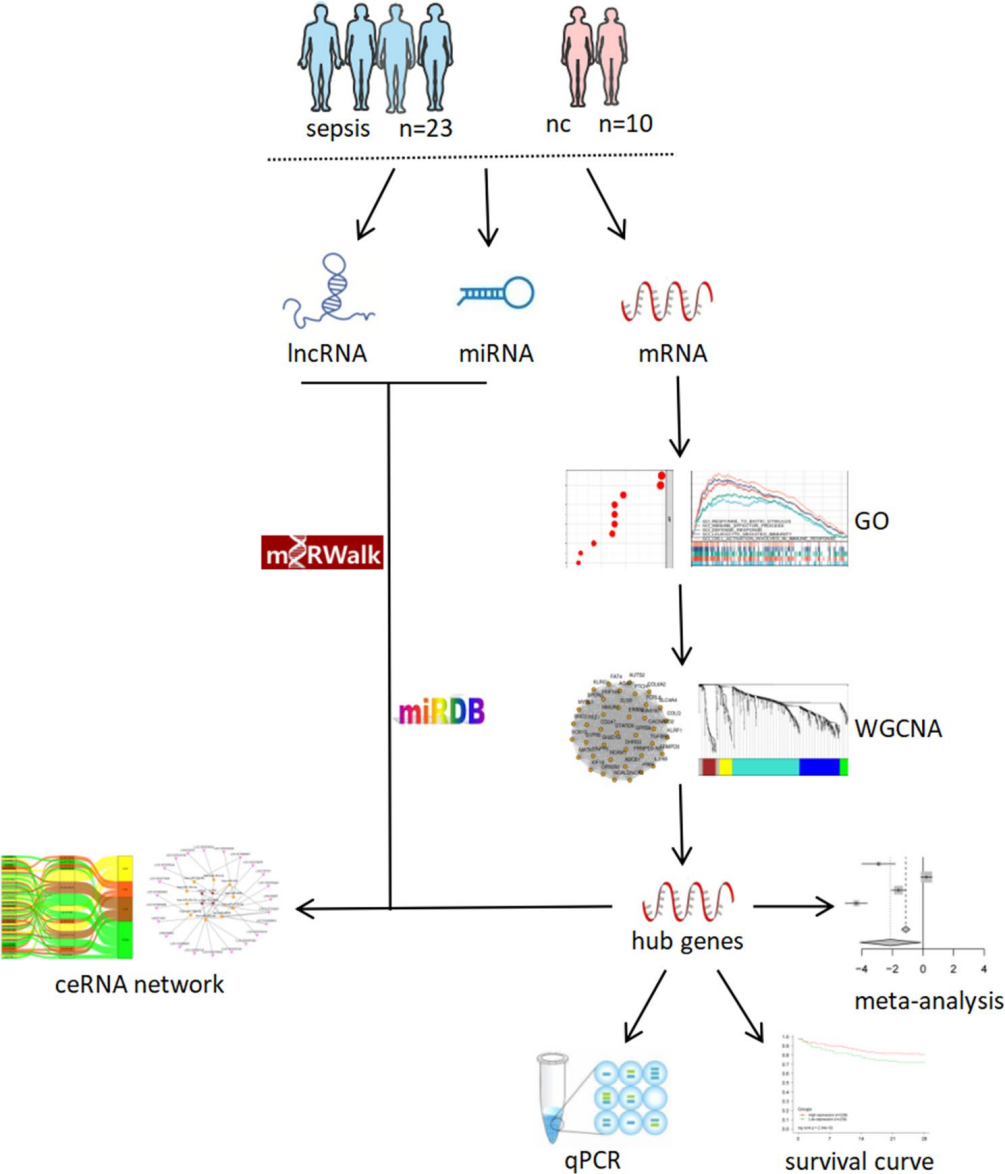
Whole ceRNA network framework based on RNA-seq data of patients with sepsis and normal control

This study adopted high-throughput sequencing technique to perform RNA-seq analysis in peripheral blood samples from sepsis patients (n = 23) and healthy people (n = 10). Additionally, bioinformatics analysis was conducted to identify potential molecular targets of survival significance, combining differential expression analysis, functional annotation and co-expression analysis. A ceRNA regulatory network was accordingly constructed, aiming to guide further research on prognosis of sepsis and provide a new thought for clinical diagnosis and treatment in the future. Workflow of the project is displayed in Fig. 1.

## Materials and methods

### Subjects and blood sampling

Consecutive cases of sepsis admitted to the Emergency Intensive Care Unit (EICU) of the Affiliated Hospital of Southwest Medical University between January 2019 and December 2019 were initially enrolled. Inclusion criteria: (1) In accordance with the Sepsis 3.0 Criteria (Infection + ΔSOFA score ≥ 2) jointly released by Society of Critical Care Medicine (SCCM) and European Society Intensive Care Medicine (ESICM) in 2016; (2) 16 ≤ age ≤ 65; (3) Written informed consent. Patients would be excluded if they previously had organ failure, immune system disease, or hematologic disease. Pregnant or lactating women were also excluded. Eventually, a total of 23 cases of sepsis were selected. Control healthy subjects (n = 10) were recruited at the same hospital, who underwent routine medical check-ups during the same period. Peripheral blood sampling was performed within 24 h after admission in sepsis patients, and control blood samples were obtained from the healthy subjects. All subjects signed informed consent. The study protocol was reviewed and approved by the ethics committee of the Affiliated Hospital of Southwest Medical University (Ethical Approval No. ky2018029). The Registration Number was ChiCTR1900021261.

### RNA-seq analysis

Peripheral blood cells were extracted and digested with Trizol to obtain total RNA. The total RNA was qualitatively and quantitatively analyzed using the Nano Drop and Agilennt 2100 bioanalyzer, respectively. Raw Reads(lncRNA/mRNA,miRNA) were filtered by the SOAPnuke (https://github.com/BGI-flexlab/SOAPnuke) [14], and the Clean Reads obtained were saved in the FASTQ format. Subsequently, the Clean Reads were aligned to the reference genome by HISAT2 software (v2.0.4) [15]. Fusion genes and differentially spliced genes (DSGs) were tested using the Ericscript (v0.5.5) [16] and rMATS (V3.2.5) [17], respectively. Finally, the Clean Reads were aligned to the genome assembly by Bowtie2 software (v2.2.5) [18]. RSEM (v1.2.12) [19] was used to calculate gene expression level.

### Screening of differentially expressed RNAs (DERs)

Data filtering and normalization were completed using the online iDEP93 platform (http://bioinformatics.sdstate.edu/idep/) [20, 21], followed by principal component analysis (PCA). PCA is a quantitatively rigorous method that clusters large amount of gene expression data into several principal components orthogonal to each other via dimensionality reduction, which helps find out the outliers and identify samples of high similarity. Differential expression analysis and statistical testing were performed using DESeq2 software. Differentially expressed mRNAs, miRNAs and lncRNAs (DEmRNA, DEmiRNA, DElncRNA) meeting |Fold Change [FC]| ≥4 and False Discovery Rate (FDR) < 0.01 were screened out.

### GO annotation and GSEA functional clustering

Gene Ontology (GO) annotations are statements about the function of a particular gene that describe Biological Process (BP), Cellular Component (CC) and Molecular Function (MF) [22]. Here, GO annotation was performed using the R4.0.5 (p < 0.05) to view the functional enrichment of the DEmRNAs. Gene Set Enrichment Analysis (GSEA) aims at showing the distribution of a given gene set in a prior defined set of genes correlated with the phenotypic class distinction to judge on their contribution to the phenotypes. Here, GSEA was performed using the R3.6.3, and the significance threshold was set as FDR < 0.25 and p.adjust < 0.05.

### WGCNA and identification of core genes

Weighted correlation network analysis (WGCNA) can be used to find clusters (modules) of genes highly correlated in expression pattern during the same physiological process or in different tissues, and the genes in the same cluster (module) are believed to be functionally similar or correlated. This method can help predict the function of a new gene or RNA [23, 24]. Here, we used iDEP93 online platform to calculate soft threshold and select modules that express consistent trends. Then a WGCNA network was constructed with this modules of genes to screen for sepsis core genes.

### Meta-analysis

Meta-analysis was devised to evaluate the expression pattern of the core genes in various datasets based on multiple microarray datasets from the GEO database (https://www.ncbi.nlm.nih.gov/geo/), including GSE28750 [25], GSE54514 [26], GSE95233 GEO Accession viewer (nih.gov), GSE6535 [27], GSE63042 [28], GSE74224 [29], GSE67652 [30] and GSE12624 GEO Accession viewer (nih.gov), using the R4.0.5 package “meta” for Meta-analysis [31]. All data were analyzed in sepsis versus normal and sepsis versus systemic inflammatory response syndrome (SIRS).

### Survival analysis

Data from a public dataset GSE65682 [32] were downloaded to explore the prognostic value of the core genes in sepsis. The GSE65682 dataset contains 478 peripheral blood samples from sepsis patients, together with gene expression profile and clinical prognostic data. Survival analysis was performed using Graphpad prism7, and p < 0.05 in log-rank test was defined as having statistical significance.

### Cell culture and sepsis modeling

Human monocytic leukemic cell line THP-1 was selected to identify the expression trend of the core genes in sepsis and perform further in vitro experiment. THP-1 cells were cultured in complete culture medium containing 10% fetal bovine serum (FBS) in an incubator with 5% CO_2_ at 37 ℃. Cell concentration was adjusted to 50 ng/ml by addition of phorbol myristate acetate (PMA). The monocytes were then induced to convert into adherent macrophages. After 48 h, the supernatant was discarded and cells were collected. The cells were then cultured for 24 h with 2 ml 10% FBS-supplemented medium free of Penicillin-Streptomycin Solution. Lipopolysaccharide (LPS) (100 ng/ml) was subsequently added to induce sepsis for 6 h. Culture medium of the control cells was changed at the same time point without any other treatment.

### RT-qPCR

PCR primers were designed by the PrimerBank database (https://pga.mgh.harvard.edu/primerbank/) [33]. Detailed primer sequences were as below: CD247 Forward GCCAGAACCAGCTCTATAACG, Reverse GGCCACGTCTCTTGTCCAA; IL1R2 Forward ATGTTGCGCTTGTACGTGTTG, Reverse CCCGCTTGTAATGCCTCCC; IL2RB Forward CTGCTTCACCAACCAGGGTTA, Reverse GGGGTCGTAAGTAAAGTACACCT; TGFBR3 Forward TGGGGTCTCCAGACTGTTTTT, Reverse CTGCTCCATACTCTTTTCGGG. Total RNA in cells was extracted using the RNA extraction kit (Tiangen, China, DP419), and the concentration and purity were measured with a spectrophotometer. Complementary DNA (cDNA) of the RNA was synthesized using a RT kit (TOYOBO, China, FSQ-201). RT-qPCR was then performed with the SYBR Green kit (TOYOBO, China, QPK-201), and melting curves were generated. The results were analyzed in 2^-∆∆Ct.

### Statistical analysis

Statistical analysis and figure processing were conducted using the R3.6.3 software. Continuous data of normal distribution were expressed at mean ± standard deviation, and the data that did not conform to normal distribution were in the form of median and quartiles. Comparison between sepsis and control groups was completed with independent-sample t test upon data meeting normal distribution and homogeneity of variance.

### Construction of a ceRNA regulatory network

Between-group correlation was analyzed using the OmicShare cloud platform (https://www.omicshare.com/). DEmiRNAs and DElncRNAs which are significantly negatively and positively associated with the DEmRNAs (p < 0.05) were screened out, respectively. miRNAs having binding sites on the 3’UTR of the core mRNAs were predicted using the miRWalk database (http://mirwalk.umm.uni-heidelberg.de/) [34, 35], and then intersected with the DEmiRNAs of significantly negative correlation. Similarly, miRNAs having binding sites on the DElncRNAs of significantly positive correlation with the key mRNAs were predicted using the miRDB database (http://mirdb.org/custom.html) [36], and then intersected with the overlapped miRNAs. The final mRNAs, miRNAs and lncRNAs screened out were projected on the OmicShare cloud platform to establish a ceRNA regulatory network.

## Results

### Demographic and clinical characteristics

There were 23 sepsis patients and 10 healthy people included in this project. Demographic and clinical characteristics of the subjects included gender, age, Sequential Organ Failure Assessment (SOFA) score, Glasgow Coma Scale (GCS) score, total white blood cell (WBC) count, neutrophil count, total bilirubin, urea, and serum creatinine (Table 1). Comparatively, the SOFA score, total WBC and neutrophil count, urea level were higher while the GCS score was lower in the sepsis group versus control group, which were statistically significant (p < 0.05).

**Table 1:** Demographic and clinical data of subjects (m ± sd). Gender, age, SOFA score, Glasgow Coma Scale (GCS) score, total white blood cell (WBC) count, neutrophil count, total bilirubin, urea, and serum creatinine

Clinic items	Sepsis(n = 23)	NC(n = 10)	P
Gender(F/M)	9/14	4/6	-
Age(years)	58.09 ± 2.365	51.7 ± 3.685	0.1503
SOFA	5.87 ± 0.6328	0 ± 0	<0.0001
GCS	11.3 ± 0.8303	15 ± 0	0.0067
WBC(×109)	13.57 ± 2.004	6.364 ± 0.5525	0.0262
NEU(×109)	11.82 ± 1.812	3,819 ± 0.4262	0.0073
TBIL	51 ± 21.47	14.3 ± 1.433	0.2729
Urea	9.654 ± 1.342	5.304 ± 0.4908	0.0446
Cre	123.9 ± 32.78	67.67 ± 3.844	0.2718

### Screening of DERs

**Fig. 2:**
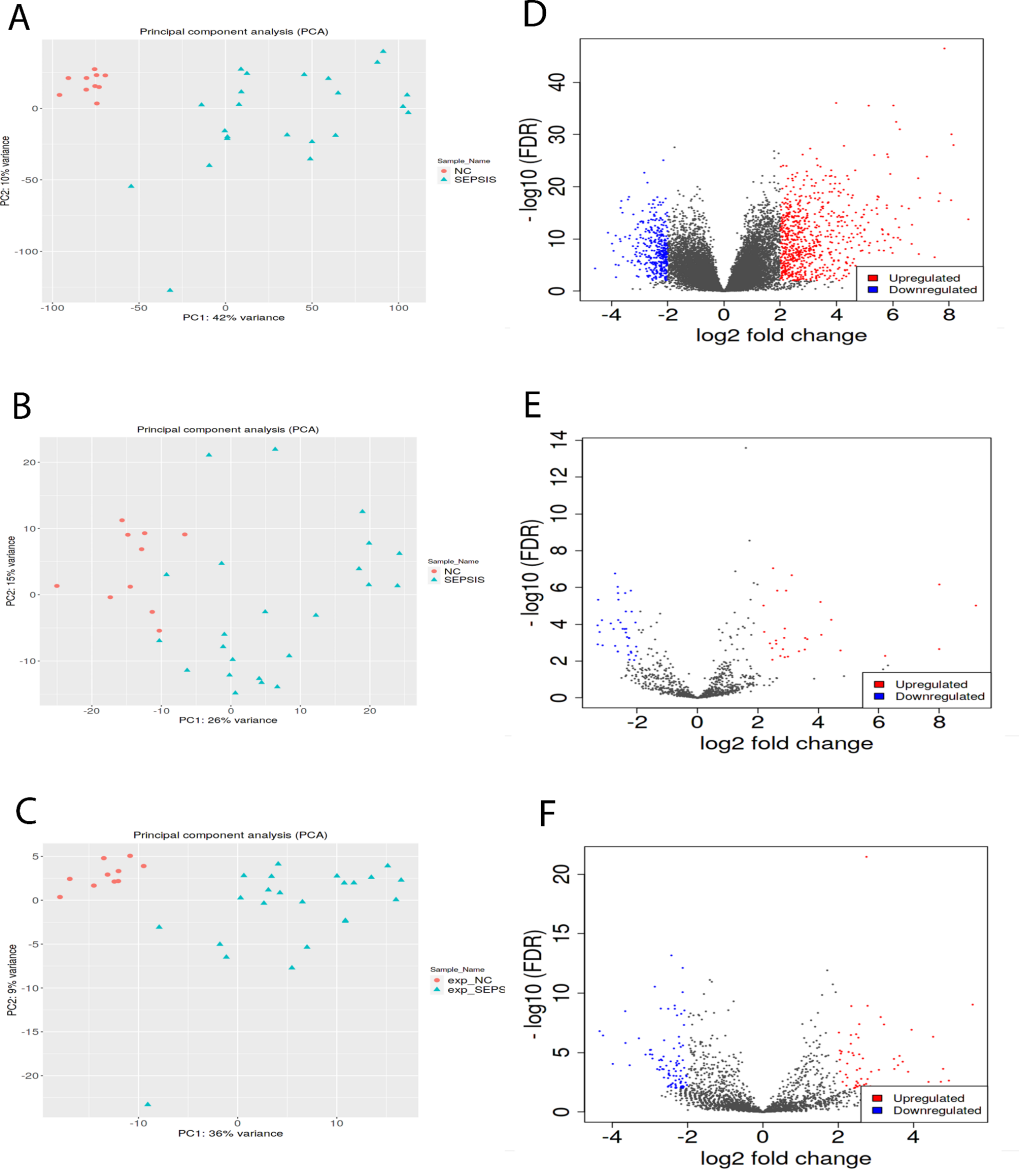
Screening of differentially expressed RNAs. (A-C) PCA analysis in mRNA (A), miRNA (B) and lncRNA (C). Red for normal control and blue for sepsis. (D-F) Volcano Plots of DEmRNA (D), DEmiRNA (E) and DElncRNA (F). Red for up-regulated genes, blue for down-regulated genes, and black for genes with no differential expression. The X-axis represents fold change (FC) and the Y-axis represents false discovery rate (FDR)

Sequencing data of mRNA, miRNA and lncRNA were subjected to PCA and two PCs were obtained, it indicates that the data were comparable between sepsis group and normal group (Fig. 2A-C). Differential analysis was conducted in the PCs, and DEGs that met |FC| ≥4 and FDR < 0.01 were selected, including 1,044 DEmRNAs (688 up-regulated and 356 down-regulated), 66 DEmiRNAs (29 up-regulated and 37 down-regulated) and 155 DElncRNAs (61 up-regulated and 94 down-regulated) (Fig. 2D-F).

### GO annotation and GSEA functional enrichment

**Fig. 3:**
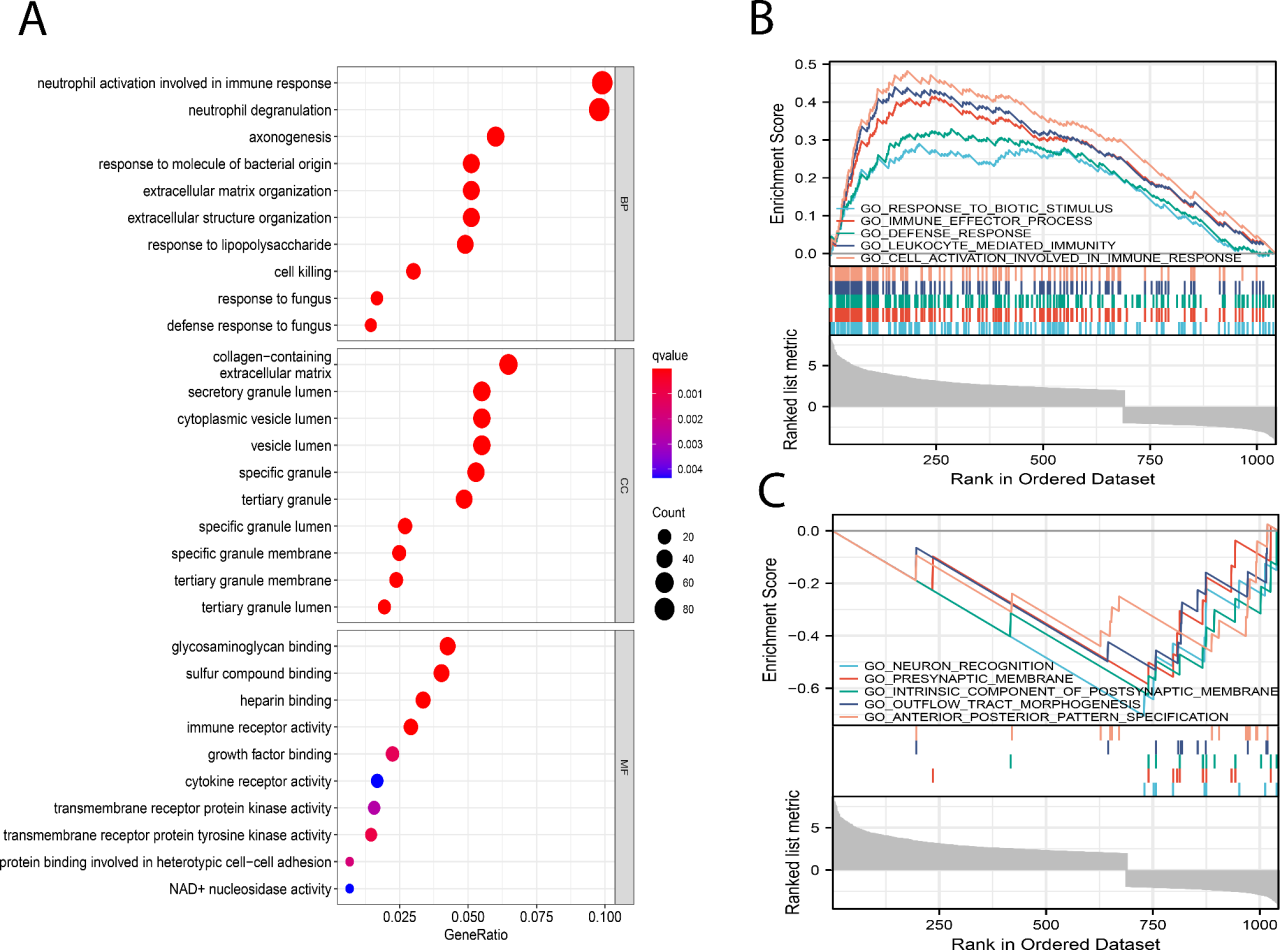
GO annotation and GSEA functional clustering. (A) GO annotations for DEmRNAs from Biological Process (BP), Cellular Component (CC) and Molecular Function (MF). The colors from blue to red correspond to the q value from highest (blue) to lowest (red). The size of circles represents the number of genes enriched in the term. (B-C) GSEA functional clustering analysis. Upper part: Normalized enrichment score (NES), which indicates the enrichment of genes in a given gene set toward the up-regulated (positive NES, peak on the left) or down-regulated (negative NES, peak on the right) end of a pre-defined gene set correlated with phenotypes in sepsis; Middle part: Each gene of the gene set is represented by a vertical line and the lines (genes) before the peak belong to the leading edge subset and contribute to the most to the phenotype; Lower part: Gene list rank after normalization

GO function enrichment analysis was performed and revealed the most enriched GO terms of the DEmRNAs, including neutrophil activation involved in immune response, neutrophil degranulation, axonogenesis, response to molecule of bacterial origin, extracellular matrix organization, extracellular structure organization, response to lipopolysaccharide, cell kllig (Fig. 3A). By the GSEA clustering analysis, the differentially up-regulated genes were significantly enriched in pathways involved in response to biotic stimulus, immune effector process, defense response, leukocyte mediated immunity, cell activation involved in immune response, and the differentially down-regulated genes were highly activated in pathways associated with neuron recognition, presynaptic membrane, intrinsic component of postsynaptic membrane, outflow track morphogenesis, anterior posterior pattern specification (Fig. 3B-C). Collectively, the DEmRNAs are mainly involved in biological processes associated with inflammatory response and immune regulation.

### WGCNA

**Fig. 4:**
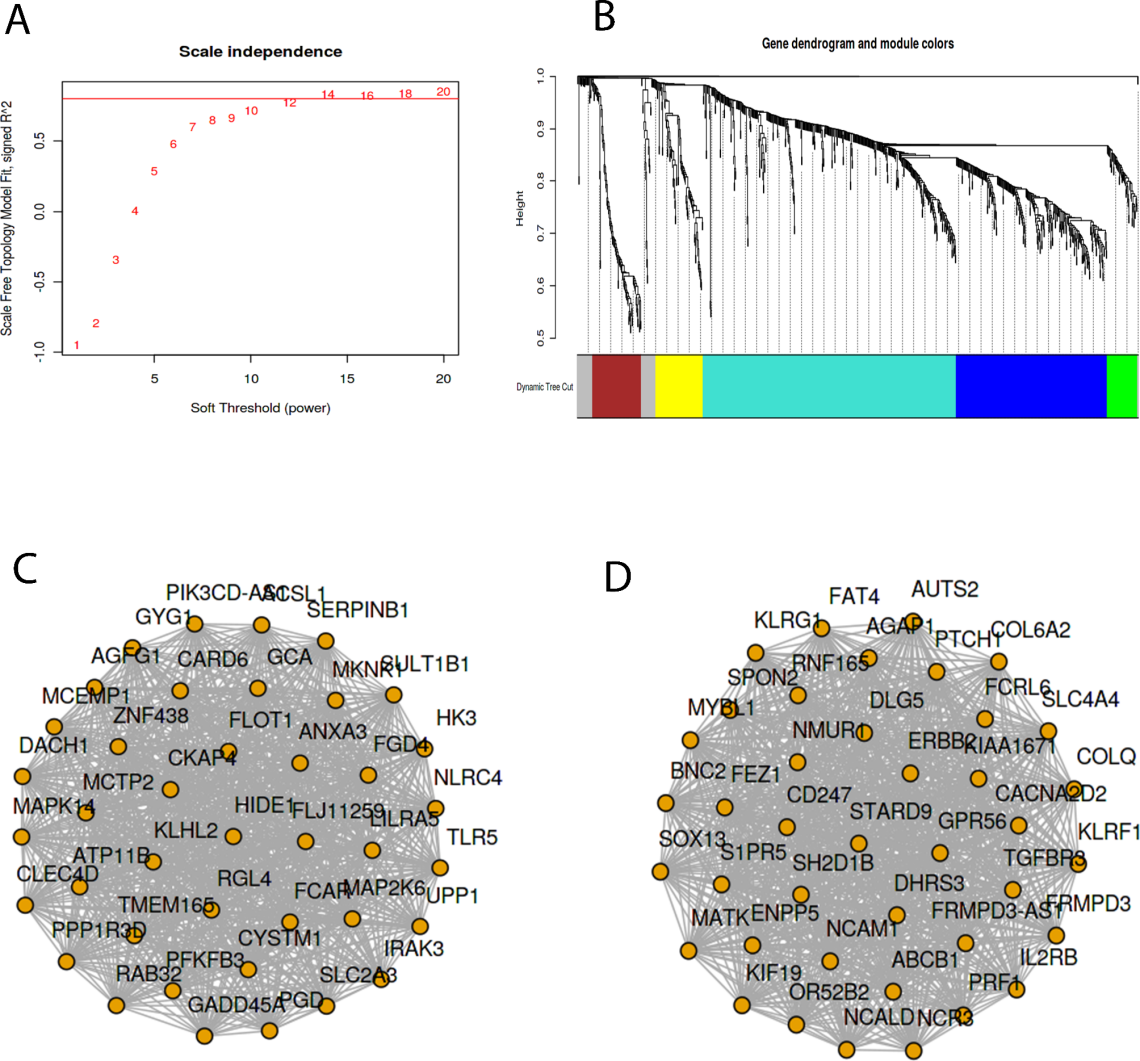
WGCNA. (A) Soft threshold value = 12. (B) Modules based on the co-expression topological overlap of mRNA in different colors (Module size > 50). The Blue and Green modules were found to be highly correlated with clinical traits in sepsis. (C-D) The top 40 genes in the Blue (C) and Green (D) modules

Modules at a soft threshold of 12 and size > 50 were obtained by WGCNA and the Blue and Green modules were found to be highly associated with clinical phenotypes in sepsis (Fig. 4A-B). S1PR5, GNLY, CD247, KLRG1, IL-1R2, AUTS2, IL-2Rβ, ARG1, TGFBR3, TLR5 and PRF1 in the top 80 genes in the two modules were located toward the center of the co-expression network, and CD247, IL-2Rβ, TGF-βR3 and IL-1R2 were identified as core genes in sepsis (Fig. 4C-D).

### Meta-analysis

**Fig. 5:**
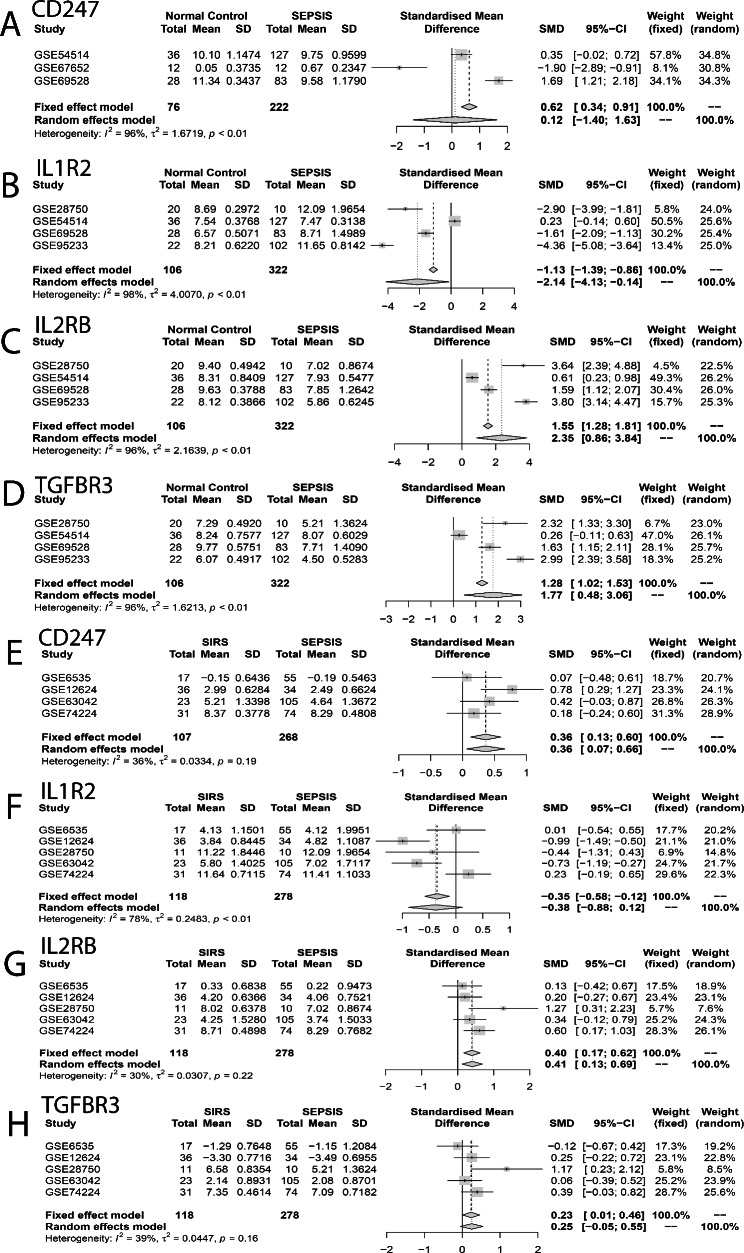
Meta-analysis. (A-D) Meta-analysis for expression of CD247, IL-1R2, IL-2Rβ and TGF-βR3 in the sepsis group versus the normal group in GSE28750, GSE54514, GSE69528, GSE95233 and GSE67652 datasets. IL-1R2 was up-regulated, while IL-2Rβ and TGF-βR3 were down-regulated in sepsis; CD247 marginally varied between the two groups. (E-H) Meta-analysis for expression of CD247, IL-1R2, IL-2Rβ and TGF-βR3 in the sepsis group versus the SIRS group in GSE28750, GSE6535, GSE63042, GSE74224 and GSE12624 datasets. CD247, IL-2Rβ and TGF-βR3 were down-regulated in sepsis versus SIRS and IL-1R2 marginally varied between the two groups. A random-effects model was used when the heterogeneity P value was < 0.05, otherwise, a fixed-effects model was used

Expression of CD247, IL-2Rβ, TGF-βR3 and IL-1R2 was analyzed in multiple microarray datasets by a meta-analysis. When comparing the sepsis patients with normal individuals, IL-1R2 was up-regulated while IL-2Rβ and TGF-βR3 were down-regulated. No statistically significant difference in CD247 expression was noted between the two groups (Fig. 5A-D). When comparing the sepsis patients with SIRS cases, CD247, IL-2Rβ and TGF-βR3 were down-regulated significantly but IL-1R2 marginally varied (Fig. 5E-H).

### Survival analysis

**Fig. 6:**
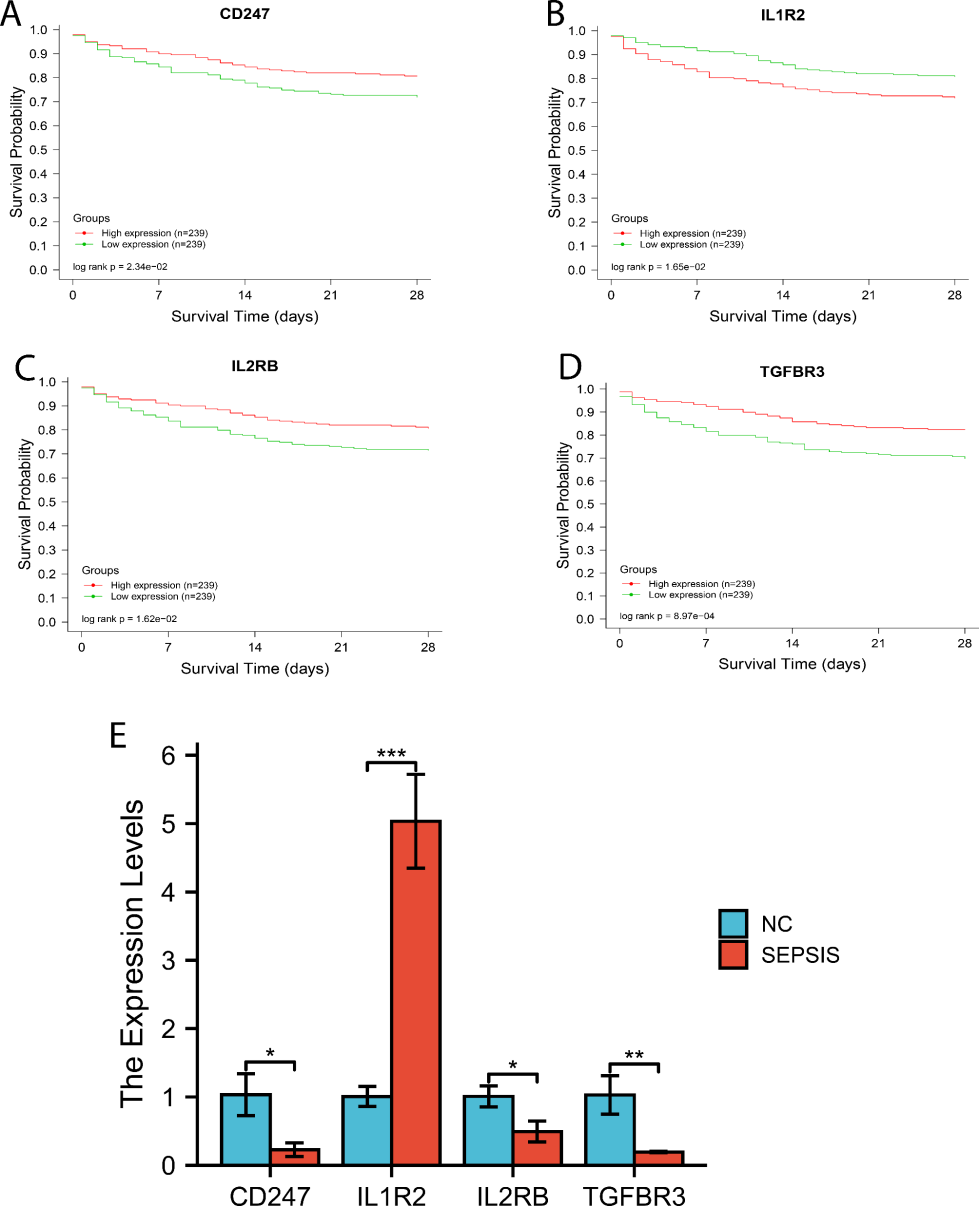
Survival analysis and RT-qPCR. (A-D) Survival analysis of CD247 (A), IL-1R2 (B), IL-2Rβ (C) and TGF-βR3 (D) for 28-day survival in patients with sepsis. CD247, IL-2Rβ and TGF-βR3 were positively correlated with survival while IL-1R2 was negatively correlated (p < 0.05). (E) RT-qPCR performed to measure the expression of the four core genes in a cellular model of sepsis. Blue for control and red for sepsis. *, p < 0.05; **, p < 0.01; ***, p < 0.001

Survival significance of CD247, IL-2Rβ, TGF-βR3 and IL-1R2 was assessed in the GSE65682 dataset. As shown in Fig. 6A-D, expression of CD247, IL-2Rβ and TGF-βR3 was positively associated with the survival in patients with sepsis, and expression of IL-1R2 was negatively associated (p < 0.05). The four genes were prognostic for the survival in sepsis and might be new research targets. The GSE datasets used for Meta-analysis and Survival analysis are presented in Table 2.

**Table 2:** Details of GSE datasets for Meta-analysis and Survival analysis

GSE datasets	Organism	Platform	Number of samples
GSE28750	Blood of Homo sapiens	GPL570	41
GSE54514	Blood of Homo sapiens	GPL6947	163
GSE69528	Blood of Homo sapiens	GPL10558	138
GSE95233	Blood of Homo sapiens	GPL570	124
GSE67652	Blood of Homo sapiens	GPL16699	24
GSE6535	Blood of Homo sapiens	GPL4274	72
GSE63042	Blood of Homo sapiens	GPL9115	129
GSE74224	Blood of Homo sapiens	GPL5175	105
GSE12624	Blood of Homo sapiens	GPL4204	70
GSE65682	Blood of Homo sapiens	GPL13667	802

### RT-qPCR

In vitro experiment was performed to test expression of CD247, IL-2Rβ, TGF-βR3 and IL-1R2 in human THP-1 cells of sepsis. By independent-sample t test, CD247 (-0.805 [-1.323 - -0.288]; t = -4.323, p = 0.012), IL-2Rβ (-0.514 [-0.862 - -0.166]; t = -4.102, p = 0.015) and TGF-βR3 (-0.835 [-1.288 - -0.382]; t = -5.120, p = 0.007) were significantly down-regulated in sepsis cells versus normal cells, while IL-1R2 (4.028 [2.904–5.153]; t = 9.950, p = 0.001) was reversely up-regulated. All the differences were statistically significant (p < 0.05) (Fig. 6E).

### ceRNA regulatory network construction

**Fig. 7:**
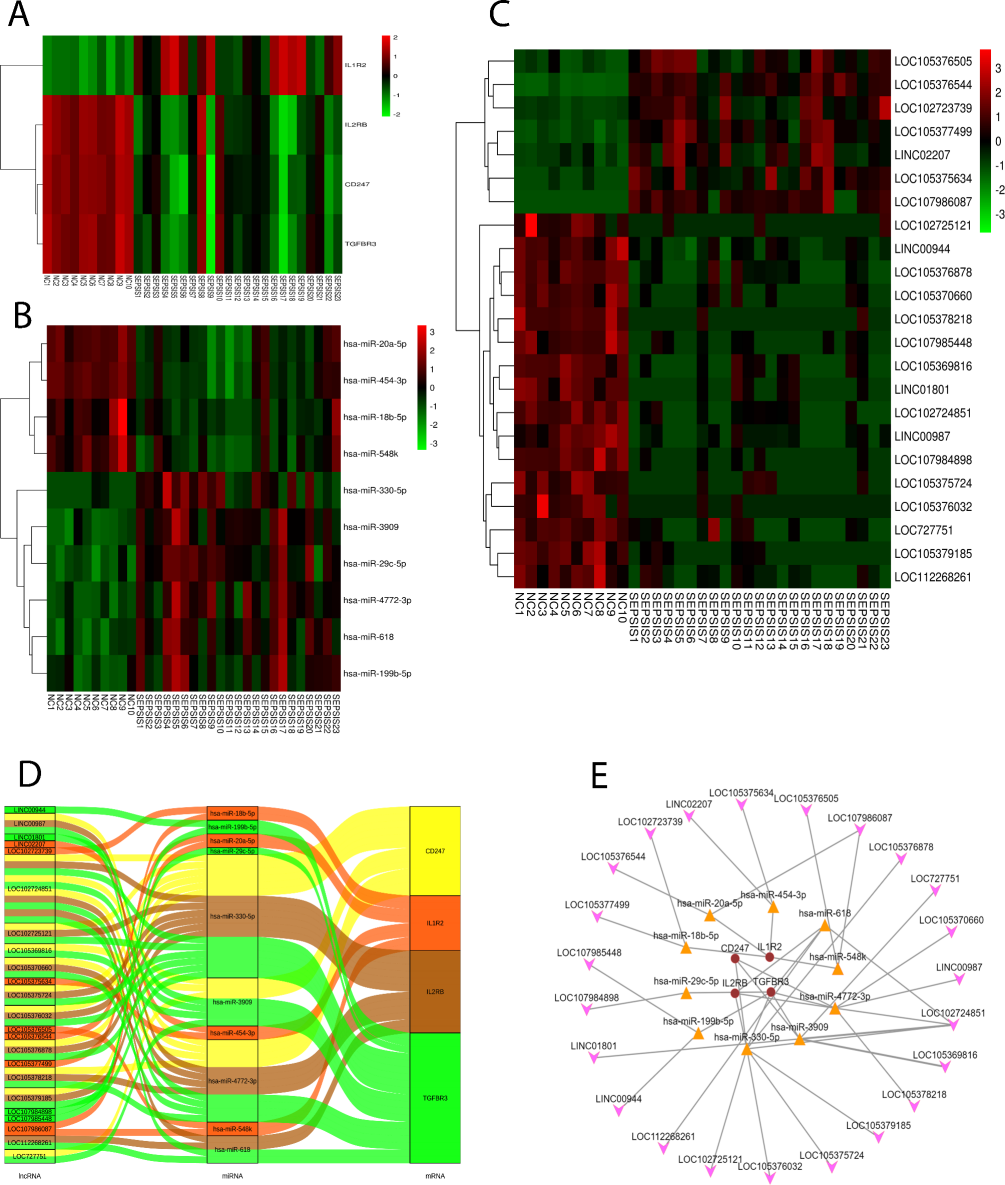
Heatmap and ceRNA regulatory network. (A-C) Heatmaps for 4 mRNAs (A), 10 miRNAs (B) and 23 lncRNAs (C). Red for up-regulated genes and green for down-regulated genes. (D-E) Sankey diagram (D) and directed network graph (E) established by the 4 mRNAs, 10 miRNAs and 23 lncRNAs (Concave quadrilateral for lncRNA, triangle for miRNA and circle for mRNA).

Following correlational analysis and molecular interaction prediction, 10 miRNAs and 23 lncRNAs highly correlated with the 4 core genes (CD247, IL-2Rβ, TGF-βR3 and IL-1R2) were obtained. Heatmaps of the mRNAs, miRNAs and lncRNAs were made as shown in Fig. 7A-C. A ceRNA regulatory network based on the lncRNA-miRNA-mRNA pairs was established. Sankey diagram and directed network graph were accordingly plotted (Fig. 7D-E) (Table 3). LncRNAs (LOC105376878, LOC727751, LOC105370660, LINC00987, LOC102724851, LOC105369816, LOC105378218, LOC105379185, LOC105375724, LOC105376032, LOC102725121, LOC112268261, LINC00944, LINC01801, LOC107984898, LOC107985448, LOC105377499, LOC105376544, LINC02207, LOC102723739, LOC105376505, LOC105375634, LOC107986087) and miRNAs (hsa-miR-330-5p, hsa-miR-3909, hsa-miR-4772-3p, hsa-miR-618, hsa-miR-199b-5p, hsa-miR-29c-5p, hsa-miR-18b-5p, hsa-miR-20a-5p, hsa-miR-454-3p, hsa-miR-548k) were found to be potentially involved in regulation of prognosis in sepsis.

**Table 3:** ceRNA regulatory network composed of 23 lncRNAs, 10 miRNAs and 4 mRNAs

Type		Hub RNA		
mRNA	CD247	IL1R2	IL2RB	TGFBR3
miRNA	hsa-miR-330-5p hsa-miR-3909 has-miR-4772-3p	hsa-miR-18b-5p hsa-miR-20a-5p hsa-miR-454-3p hsa-miR-548k	hsa-miR-330-5p hsa-miR-4772-3p hsa-miR-618	hsa-miR-330-5p hsa-miR-199b-5p hsa-miR-3909 hsa-miR-4772-3p hsa-miR-618 hsa-miR-29c-5p
lncRNA	LOC105376878 LOC727751 LOC105370660 LINC00987 LOC102724851 LOC105369816 LOC105378218 LOC105379185 LOC105375724 LOC105376032 LOC102725121	LOC105377499 LOC105376544 LINC02207 LOC102723739 LOC105376505 LOC105375634 LOC107986087	LOC105376878 LOC105370660 LINC00987 LOC102724851 LOC112268261 LOC105378218 LOC105379185 LOC105375724 LOC105376032 LOC102725121	LOC105376878 LINC00944 LOC727751 LOC105370660 LINC00987 LOC102724851 LOC105369816 LINC01801 LOC107984898 LOC112268261 LOC107985448 LOC105378218 LOC105379185 LOC105375724 LOC105376032 LOC102725121

## Discussion

Despite a well understanding of the pathogenesis, mortality of sepsis remains high after treatment both domestically and abroad [37]. Therefore, early diagnosis and treatment appear to be vital important [38]. Robust diagnostic biomarkers can promote the realization of early diagnosis. In the present study, a total of 1,044 mRNAs were screened out with differential expression in sepsis versus normal samples, and enrichment analysis revealed that the DEmRNAs were mainly enriched in biological processes associated with inflammatory response, immune regulation and neutrophil activation In the meantime, 66 DEmiRNAs and 155 lncRNAs were obtained. A ceRNA regulatory network was accordingly constructed based on 23 lncRNAs, 10 miRNAs and 4 mRNAs highly correlated, and could be a potential biomarker to guide clinical diagnosis and prognosis of sepsis.

LncRNA is a class of ncRNA molecules composed of more than 200 nucleic acids. They can regulate gene expression but are incapable of coding proteins. miRNA is a short, single-stranded ncRNA at a length of 18–23 nucleic acids. miRNA could regulate gene expression via specifically binding to the 3’UTR of the downstream target mRNA. Additionally, it is involved in tumorigenesis and development by serving either an oncogene or a tumor suppressor gene. LncRNA can serve as a ceRNA to competitively bind to miRNA with downstream mRNA to promote mRNA expression and activity recovery. There are regulatory associations among lncRNA, miRNA and mRNA, which are involved in a variety of biological processes, such as cell proliferation, apoptosis, invasion and cell cycle. Dysregulation of ceRNA network may lead to incidence of multiple diseases, such as ovarian cancer [39], colorectal cancer [40], glioblastoma [41] and liver fibrosis [42]. Recent research found that miRNA could regulate the TLR4/NFκB pathway, a pathway responsible for the expression of pro-inflammatory cytokines in sepsis [43]. This infers that ceRNA may play a role in occurrence and development of sepsis.

Cluster of differentiation 247 (CD247) is an adaptor important in signal transduction mediated by T cell antigen-receptor complex and it plays a vital part in lymphocyte signaling. The non-coding sequence polymorphism of CD247 is under strict regulation and correlated with multiple immune responses and autoimmune diseases [44, 45]. Research reported that in cases of systemic lupus erythematosus (SLE), more than a half had attenuation or deficiency of CD247 expression [46]. Here, we found that patients highly expressing CD247 had a higher survival rate at 28 days. The augmentation of CD247 expression might be attributed to the binding of lncRNA with downstream hsa-miR-330-5p, hsa-miR-3909, hsa-miR-4772-3p, which concurrently advanced TCR signaling cascade reactions and assembly of T cell surface TCR/CD3 complex [47], resulting in enhanced resistance to pathogen invasion and subsequently improving the survival in patients. Our in vitro cellular experiment revealed lower expression of CD247 in sepsis versus the heathy control, which was statistically significant (p = 0.012), consistent with the RNA-seq result. This further demonstrates that the poor expression or deficiency of CD247 might lead to incidence and development of sepsis.

Interleukin-1 receptor family (IL-1R) plays a core part in immune and inflammatory responses and the members are distributed in the majority of cells of the congenital and adaptive immunities. IL-1 family members are emerging as key participants in regulating the differentiation and function of congenital and adaptive lymphoid cells. IL-1R2 is a member of the IL-1R family that acts as a negative regulator of the IL-1 system to inhibit the maturation of IL-1, isolate its activated form or impede the assembly of signal complex [48, 49]. In the current study, RT-qPCR was performed to show differential high expression of IL-1R2 in sepsis, which was statistically different with that in healthy people (p = 0.001), consistent with the RNA-seq analysis. Combining the survival and meta-analysis, we speculated that IL-1R2 expression increased in sepsis due to the binding of up-stream lncRNA with hsa-miR-18b-5p, hsa-miR-20a-5p, hsa-miR-454-3p and hsa-miR-548k, which negatively regulated IL-1 level leading to decline of the body’s anti-inflammatory capability and subsequent sepsis development and patient death.

IL-2 is a cytokine that plays a core part in infection by delivering immune signals through IL-2/IL-2R complex [50, 51]. IL-2 receptor (IL-2R) consists of three subunits: IL-2Rα, IL-2Rβ and IL-2Rγ. IL-2Rβ gene deficiency may lead to life-threatening immune dysregulation [52]. In the current study, RT-qPCR was conducted and we found that expression of IL-2Rβ remarkably decreased in sepsis as compared to that in normal people (p = 0.015), consistent with the RNA-seq analysis. We reasoned that upstream LOC105376878, LOC105370660, LINC00987, LOC102724851 competitively bound with the downstream miRNA to promote IL-2Rβ expression and the recovery of activity, which further regulated immune response and facilitated patient survival.

Transforming growth factor–β (TGF-β) is essential for organisms to maintain homeostasis and develop normally. TGF-β responsiveness and dysregulation of downstream signaling pathways might be risk factors for multiple diseases, and they may play a role in tumorigenesis, development and metastasis. TGF-β can bind to three isotype receptors of TGF-βR (TGF-βR1/2/3) with different affinities. TGF-βR3 generally shows a high expression in some tumors [53], such as endometrial cancer [54], pancreatic carcinoma [55] and cervical cancer [56]. Current studies on TGF-βR3 in sepsis are limited. We speculated the involvement of lncRNAs (LOC105376878, LINC00944, LOC727751) and miRNAs (hsa-miR-330-5p, hsa-miR-199b-5p, hsa-miR-3909) in the TGF-βR3 associated ceRNA regulatory network, which could augment the TGF-βR3 expression and in turn prolong the survival in patients of sepsis. Here, expression of TGF-βR3 profoundly decreased in a cellular model of sepsis when comparing to the normal control (p = 0.007), consistent with the RNA-seq analysis. High TGF-βR3 expression, therefore, might be conducive to prolonging the survival in patients with sepsis.

To conclude, the lncRNAs, miRNAs and mRNAs we identified here may not act in sepsis by a single mechanism, instead, by the ceRNA regulatory mechanism of interactions between RNAs or by the interactions between gene-coding proteins. Combining the RNA-seq and bioinformatics analysis, here we proposed a ceRNA regulatory network composed of 23 lncRNAs, 10 miRNAs and 4 mRNAs, which participates in the occurrence, development and prognosis of sepsis. The network could be a potential biomarker to guide further studies on clinical diagnosis and prognosis of sepsis. The current study still has some limitations. First, the sample size is small, requiring large-scale studies to validate the conclusion of the study. Second, the ceRNA regulatory network constructed here was only based on bioinformatics analysis without experimental validation. Prior to clinical application, experiments should be devised to identify the relationship between the RNAs. Third, the potential mechanism of action of the four core genes for prognosis in sepsis needs to be explored in further functional studies.

## Electronic supplementary material

Below is the link to the electronic supplementary material.

Supplementary Material 1

Supplementary Material 2

## Data Availability

We intend to share individual deidentified participant data. Peripheral blood RNA sequencing data from 23 patients with sepsis and 10 normal controls are available in the China National GeneBank DataBase (CNGBdb) and can be found below: https://db.cngb.org/, under the accession: CNP0002611, you can access it now and it’s valid forever.
