# Supplementary material for: Screening of core genes prognostic for sepsis and construction of a ceRNA regulatory network
Source: BMC Med Genomics. 2023 Feb 28;16:37. doi: 10.1186/s12920-023-01460-8 (PMC9976425; doi:10.1186/s12920-023-01460-8)
Supplement: Supplementary file 1 — Supplementary Material 1 [file 12920_2023_1460_MOESM1_ESM.docx]

**Availability of Data and Materials**

We intend to share individual deidentified participant data. Peripheral blood RNA sequencing data from 23 patients with sepsis and 10 normal controls are available in the China National GeneBank DataBase (CNGBdb) and can be found below: https://db.cngb.org/, under the accession: CNP0002611, you can access it now and it’s valid forever.
