# Supplementary material for: Screening of core genes prognostic for sepsis and construction of a ceRNA regulatory network
Source: BMC Med Genomics. 2023 Feb 28;16:37. doi: 10.1186/s12920-023-01460-8 (PMC9976425; doi:10.1186/s12920-023-01460-8)
Supplement: Supplementary file 2 — Supplementary Material 2 [file 12920_2023_1460_MOESM2_ESM.docx]

**Declaration**

**Ethics approval and consent to participate**

The study was conducted in strict accordance with the rules of the Declaration of Helsinki.The study protocol has been approved by the ethics committee of the Affiliated Hospital of Southwest Medical University (Ethical Approval No. ky2018029). The Registration Number was ChiCTR1900021261.

**Consent for publication**

If any individual or any person's data in this article needs the consent of others to be published, all You need the consent of others before you declare it here.

**Informed consent**

Informed consent was obtained from all individuals included in this study.

**Competing interests**

Authors state no conflict of interest.

**Author contributions**

LCW, QZ, YL, HMC, CYH ,LSL,designed the study. QZ, LCW, YL and HMC performed the bioinformatics analysis and interpretation of the data. QZ, LCW wrote the manuscript. CYH revised the manuscript and gave final approval of the version to be published. All authors read and approved the final manuscript. All authors have accepted responsibility for the entire content of this manuscript and approved its submission.

**Fund support**

Department of Science and Technology of Sichuan Province, 2019JDPT0003.

**Acknowledgements**

We thank BGI for instructing RNA sequencing.
